# Observation of non-recommended (harmful) intrapartum practices among obstetric care providers in public hospitals in southern Ethiopia, 2023

**DOI:** 10.1371/journal.pgph.0003375

**Published:** 2024-07-11

**Authors:** Dagne Deresa Dinagde, Shambel Negesa Marami, Gizu Tola Feyisa, Hana Tadesse Afework, Nikodimos Eshetu Dabe, Habtamu Wana Wada, Teklemariam Gultie

**Affiliations:** 1 Department of Midwifery, College of Health Sciences, Mattu University, Mettu, Ethiopia; 2 Department of Midwifery, College of Health Sciences, Mizan-Tepi University, Mizan, Ethiopia; 3 Department of Biomedical Sciences, College of Health Sciences, Mizan-Tepi University, Mizan, Ethiopia; 4 Department of Midwifery, College of Health Sciences, Arba Minch University, Arba Minch, Ethiopia; The University of Newcastle Australia: University of Newcastle, AUSTRALIA

## Abstract

The majority of developing countries do not follow the WHO’s emphasis on replacing harmful and ineffective traditional practices with evidence-based clinical treatment. In these countries, harmful or ineffective practices are routinely used as part of routine care during labor and delivery, while beneficial procedures are not used for the majority of laboring mothers. However, it is critical to use evidence-based practices while giving therapy since they improve care quality, save costs, increase patient and family happiness, and promote professional progress. To assess the magnitude of non-recommended (harmful) intrapartum practices among obstetric care providers in public hospitals in southern Ethiopia, 2023. An institution-based cross-sectional study was conducted from January 30, 2023, to February 30, 2023, in public hospitals in the Gamo and Gofa zones. An observational checklist and a self-administered questionnaire were used to gather data. Using odds ratio of 95% C, bivariate and multivariable logistic regression was used to discover factors related with the outcome variable during data analysis using SPSS version 27. A P-value of less than 0.05 and I were regarded as statistically significant. The magnitude of harmful intrapartum practice was 60.6% (95% CI: 53.25–68.5). Lack of internet access (AOR = 10.1, 95% CI: 4.93–21.1), a few years of work experience (AOR = 6.21, 95% CI: 3.1–12.5), and not being trained on evidence-based intrapartum practices (AOR = 4.01, 95% CI: 1.94–7.95) were statistically significant with harmful intrapartum practices. Evidence-based practice can be improved by promptly providing obstetric care providers with ongoing training and standards for intrapartum care.

## Introduction

"Intra-partum care" is the phrase used to describe the care given throughout the first, second, third, and fourth stages of labor, which usually last for one to two hours following the delivery of the placenta [1]. It is critical to eliminate intrapartum deaths through enhanced intra-facility care [2]. The emphasis on improvement has been on quality of care in order to lower avoidable death and morbidity rising from the intrapartum period in both moms and newborn babies [3].

The World Health Organization (WHO) defines evidence-based intrapartum care and practice (EBP) as the application of the best available, current, valid, and relevant evidence in clinical decision-making practice, such as research, work experience, and updated standard guidelines. EBP is regarded as a crucial element of quality care and a highly effective strategy for enhancing the quality of midwifery services [3, 4].

The majority of developing countries do not follow the WHO’s emphasis on replacing harmful and ineffective traditional practices with evidence-based clinical treatment. In these countries, harmful or ineffective practices are routinely used as part of routine care during labor and delivery, while beneficial procedures are not used for the majority of laboring mothers [5, 6]. However, it is critical to use evidence-based practices while giving therapy since they improve care quality, save costs, increase patient and family happiness, and promote professional progress [7].

The results of the survey show that 810 women die per day from complications related to pregnancy and childbirth, with the majority of these deaths occurring in low- and middle-income countries (LMICs) [8]. Moreover, every year, 2.1 million stillbirths and 2.6 million neonatal fatalities occur worldwide; about half of these deaths are related to complications during childbirth in settings with limited resources [2].

With improved treatment, a significant amount of perinatal deaths that transpire during the intrapartum phase can be prevented; perinatal mortality dropped by 22% among individuals who followed evidence-based practice [2, 9]. Globally, only 74% of developing nations adhered to EBP. Additionally, a study conducted in Ethiopia revealed that only 38.2% of obstetric care professionals used evidence-based intrapartum practice [10].

Age, gender, year of experience, profession, favorable attitude, adequate knowledge, and on-the-job training on EBP were factors associated with evidence-based practice among obstetric care providers in the world [11–14]. With improved treatment, a significant amount of perinatal deaths that transpire during the intrapartum phase can be prevented; perinatal mortality dropped by 22% among individuals who followed evidence-based practice [2, 9]. Globally, only 74% of developing nations adhered to EBP. Additionally, a study conducted in Ethiopia revealed that only 38.2% of obstetric care professionals used evidence-based intrapartum practice [10].

Age, gender, year of experience, profession, favorable attitude, adequate knowledge, and on-the-job training on EBP was factors associated with non-recommended intrapartum practice among obstetric care providers in different literatures [11, 13, 14]. However, there has been no study conducted on non-recommended (harmful) intrapartum practices in Ethiopia. As a result, the purpose of this study was to determine the extent of non-recommended intrapartum care practice and associated factors among obstetric care providers in government hospitals in the Gamo and Gofa zones in 2023.

## Methods and materials

### Study design and setting

An institution-based cross-sectional study was conducted from January 30, 2023, to February 30, 2023, in public hospitals in the Gamo and Gofa zones. One of Ethiopia’s regions, the Southern Nation Nationalities and People’s Regional State (SNNPR), is home to several zones. The Central Statistical Agency of Ethiopia (CSA) performed a census in 2007 that indicates 1,659,310 people live in these zones overall. In terms of health care coverage, Gamo Zone has 63 public health facilities, of which 57 are health centers and 6 hospitals, namely Arba Minch General Hospital, Dilfana Primary Hospital, Kamba Primary Hospital, Gerese Primary Hospital, Chencha Primary Hospital, and Selam Ber Primary Hospital. Obstetric care providers in these facilities are 606. Whereas, Gofa Zone has two hospitals named Sawla General Hospital and Laha Primary Hospital. These hospitals all offer surgical operations, prenatal care, intrapartum care, postpartum care, pediatrics, immunizations, and family planning services in addition to inpatient and outpatient medical services.

### Study population

The source population comprised all obstetric care providers working in government hospitals in the Gamo and Gofa zones, while the study population consisted of all obstetric care professionals actively employed during the data collection period.

### Eligibility criteria

This study included obstetric care professionals who had been employed by the hospital for longer than six months; however, it did not include obstetric care providers who were on maternity or annual leave at the time of data collection.

### Sample size determination

A single population proportions formula is used to determine the sample size needed for this investigation. A comparable study conducted at Amhara regional referral hospitals estimated that 61.8% of obstetricians used evidence-based intrapartum practices [10]. Alternatively, this study indirectly suggests that around 38.2% of healthcare professionals did not adhere to evidence-based practices and instead implemented non-recommended approaches, margin of error 5%, and confidence level 95%.

The required sample size (n) was calculated as follows:

$$n={\left(za\right)}^{2}\frac{p(1-p)}{d^{2}}$$

$$n={\left(1.96\right)}^{2}\frac{0.382(1-0.382)}{0.05^{2}}=363$$

With the above inputs the minimum sample size required for this study was 363. By taking 10% for non- response rate the final sample size is 400. However, due to very small amount of study population correction formula was used. Thus, data was collected from total participants of 240.

Where: n- minimum sample size

Z_α/2_- the desired level of confidence level 95% (1.96)

d- Margin of error assumed to be 5%.

P- Proportion of non-recommended intra-partum practice (38.2%)

### Sampling technique and procedure

**Fig 1:**
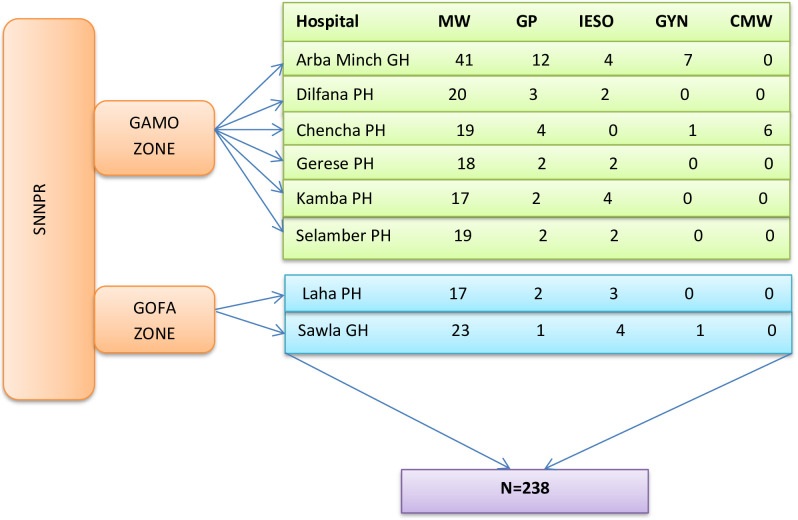
Schematic presentation of sampling of study participants in government hospitals of Gamo and Gofa zones, southern Ethiopia 2023. **Notes:-**MW- Midwife, GP- General Practitioner, IESO-Integrated Emergency Surgery Officers, SNNPR-South Nation, Nationality and Peoples Region, GYN-Gynecologist, CMW**-C**linical Midwifery, GH-General hospital, PH-Primary hospital.

Eight government hospitals are located in the Gamo and Gofa Zones. Since the total number of obstetric care providers in these hospitals is less than the total sample size determined, all obstetric care providers meeting the inclusion criteria were included in the study; all obstetric care providers in the zones are shown in **(Fig 1)** below. A total of 238 obstetric care providers were located in the hospitals and were included in the study.

### Data collection procedure

Data was collected using a semi-structured, self-administered questionnaire that was adapted from a previous study [15]. The tool contains four parts, including socio-demographic factors, individual and organizational factors, knowledge, and attitude-related questions. In addition, an observational checklist containing 21 items was adapted from “WHO recommendations on intra-partum care for a positive childbirth experience” to assess the practice [10]. Data collection was undertaken by 10 BSc midwife data collectors who were familiar with the study area. The self-administered questionnaire was collected with the help of trained midwife data collectors and one supervisor. An irreplaceable code was given for each respondent’s questionnaire. The observational data was collected using the observational checklist by the data collectors. Each obstetric care provider was observed three times by using the non-participatory observation technique during the intrapartum period to assess non recommended intrapartum practices.

### Variables of the study

#### Dependent variable

Non-recommended Intrapartum practices (Yes/No).

#### Independent variables

*Socio-demographic factors*. Age, Sex, Marital status, Profession, Educational level.

*Organizational factors*. Access to standard update, Access to computer, Access to internet, Managerial support, Regular mentoring, In-service training, and work load (number of deliveries attended per month).

*Individual factors*. Year of experience, Computer for reading, Knowledge, Attitude, Searching for Scientific Journals, Searching for Cochrane database, and searching for WHO RHL.

### Operational definition

#### Non-recommended intrapartum practices

The World Health Organization has identified certain practices as non-recommended intrapartum care. These practices include fundal pressure during the second stage of labor, routine use of chlorohexidine to clean the vagina, routine use of antibiotics for simple deliveries, routine oral or nasal suction for babies with clear amniotic fluid, interventions to speed up or stop labor before 5-cm dilatation, perineal shaving, routine amniotomy, repeated per-vaginal examination and routine or liberal episiotomy. Any of the aforementioned practices will result in the provider being assigned to non-recommended (harmful) intrapartum practices [16].

#### Evidence based intrapartum care practice

It is a collection of standard procedures that are intended to be carried out by obstetric care providers during intrapartum care and are advised by the World Health Organization and national protocols [13, 16].

#### Obstetric care providers

A certified health personnel who provide care for the woman during labor and delivery [13].

#### Adequate knowledge

Obstetricians who achieved a score higher than or equal to the mean on intrapartum care knowledge questions [10].

#### Positive attitude

Positive attitude was assigned for those scored by participants who respond above the mean of the attitude assessment questions and if below the mean they were categorized as having negative attitude [10].

#### Application of chlorohexidine for vaginal cleansing

According to WHO recommendations, chlorohexidine can be used for vaginal cleansing in specific situations. These include preoperative preparation of the vagina for gynecological or obstetric surgeries, as well as in cases of prolonged labor or ruptured membranes lasting more than 18 hours. In these situations, chlorohexidine can be used under the guidance of healthcare professionals to reduce the risk of infections. Except for cases where there are clear indications, healthcare professionals who provide this service without the presence of such indications are engaging in practices that are considered harmful [16, 17].

#### Routine oral or nasal suction

According to WHO recommendations, oral or nasal suction is not routinely recommended for babies with clear amniotic fluid. Suctioning should only be performed if there are signs of obstruction, such as meconium-stained amniotic fluid or poor respiratory effort. In these cases, healthcare professionals should assess the baby’s condition and determine the need for suctioning based on clinical judgment, not routine [18, 19].

#### Routine antibiotics administration

In line with WHO recommendations, routine use of antibiotics for simple deliveries is not advised. Antibiotics should be reserved for specific situations, such as when there is clear evidence of infection or when the mother has risk factors, like prolonged rupture of membranes or fever. It is crucial to exercise caution in the use of antibiotics to mitigate the development of antibiotic resistance. Therefore, healthcare professionals who prescribe medications without the presence of these indications are engaging in practices that are considered harmful [20].

#### Routine Amniotomy (ARM)

As per WHO recommendations, it is not advised to perform routine amniotomy (artificial rupture of membranes) during labor. Amniotomy should be reserved for specific situations, including instances of fetal distress, prolonged labor, or when the healthcare provider deems it necessary based on the unique circumstances of the laboring woman [21, 22].

#### Routine episiotomy

According to WHO recommendations, routine or liberal episiotomy is not recommended. Episiotomy should be performed selectively and only in specific situations. These may include cases where there is a need to expedite delivery due to fetal distress or when a spontaneous tear is imminent and a controlled incision may be preferred. However, routine or liberal use of episiotomy without clear indications is discouraged [23, 24].

#### Fundal pressure

Fundal pressure should not be performed and abandoned practices during the second stage of labor. Fundal pressure refers to the application of pressure on the upper abdomen to aid in the expulsion of the baby. This practice is not recommended due to the potential risks associated with it, such as uterine rupture or fetal injury [25, 26].

#### Searching for different website

Searching different websites about health sciences allows providers to explore various viewpoints and sources of information, which can provide a broader understanding of a topic. This variable was depends on participants’ report whether that they are visiting such websites to gain an understanding of evidence-based practices [13].

### Data quality control

The initial step in developing the data collection tool involved reviewing various English-language publications and utilizing readily available resources [10, 13, 27–29]. Data collectors received one day of training on administering questionnaires and observing. Two weeks prior to the real data collecting period, the pre-test is generally carried out on 5% of sampled population (obstetric care providers) that is similar to the study population rather than the study population at Wolaita Sodo University’s comprehensive specialty hospital. Based on the results of the pre-test, adjustments were made to the wording, clarity, and organization of the instrument. Following the pretest, an evaluation was conducted to assess the internal consistency and content validity of the questionnaires. It was found that Cronbach’s alpha, a measure of internal consistency, was at an acceptable level (> 0.7). Daily data entry and supervision were carried out to ensure the completeness of the surveys on actual data collection.

### Data analysis and interpretation

The data was coded, input, cleaned, and verified using Epi Data statistical software version 4.6. Subsequently, the analysis was performed using SPSS version 27. Mean and standard deviation were calculated for numerical variables to provide descriptive information. Descriptive statistics were presented using text, tables, graphs, and charts, including frequency and percentage distributions for categorical variables. An examination of association was conducted using a binary logistic regression model. The association between the independent and outcome variables was examined using bivariate analysis at a 95% confidence level. Variables that showed a p-value of less than 0.25 were included in the multivariable model to account for any potential confounders. Variables that exhibit statistical significance at a p-value of less than 0.05 were identified as contributing factors to non-recommended intrapartum care. The Hosmer and Lemeshow goodness of fit test was used to assess the model’s fitness, and the variable inflation factor (VIF) was used to assess the multi-collinearity test.

### Ethical considerations

Ethical clearance was obtained from the Arba Minch University College of Medicine and Health Science Institutional Research Ethics Review Committee with reference number of IRB/HT 1334/2022. Prior to the start of data collecting, Arba Minch University’s department of midwifery wrote an official letter to all government hospitals in Gamo and Gofa. The letter was given to the hospital’s medical directors and the leaders of the labor and delivery units. Participants were informed about the purpose of the study and the confidentiality of the information. Informed, voluntary, written, and signed consent was obtained from each participant. For this, a page of the information sheet and a page of the consent letter form were attached to each questionnaire. The names of the respondents were not recorded for the sake of confidentiality. Informed verbal consent was obtained from laboring mothers for the sake of privacy.

## Results

### Socio-demographic characteristics

A total of 231 obstetric care providers participated in this study, with a response rate of 97%. Concerning the educational level, the majority of respondents were BSc holders (56.7%), followed by diploma midwives (16.5%). The majority of the participants, 150 (65%), were females. Most care providers were 175(75.8%), midwives. 103 (44.6%) of the study participants have a monthly income of more than five thousand Ethiopian birr (**Table 1**).

**Table 1:** Socio-demographic characteristics of the study participants in government hospitals of Gamo and Gofa zone, southern Ethiopia, 2023 (n = 231).

Variable		Frequency	Percent
Age (years)	Less than 25	16	6.9
	25–30	142	61.5
	Above 30	73	31.6
Sex	Male	81	35.1
	Female	150	64.9
Marital status	Single	105	45.5
	Married	114	49.4
	Divorced	5	2.1
	Widowed	7	3.0
Educational level	Diploma	38	16.5
	BSc	131	56.7
	MSc	26	11.3
	Medical doctor	29	12.5
	Specialty	7	3.0
Profession	General practitioner	29	12.5
	Midwife	175	75.8
	IESO	20	8.7
	Obstetrician and Gynecologist	7	3.0
Monthly salary (ETB)	<5000	11	4.8
	5001–7999	117	50.6
	>8000	103	44.6

IESO- Integrated Emergency Surgical Officers

### Individual and organizational factors

More than half, 58.9% of the 231 respondents in total reported not receiving intrapartum care training while in employment. Only one-third, 34.6% of study participants had internet access. Of the 11.7% who reported having access to a computer at workplace, 77.8% of them used it for documenting patient data, whereas 11.1% of individuals used computers for reading scientific literature and digital communication, respectively (**Table 2**).

**Table 2:** Individual and organizational factors obstetric care providers in government hospitals of Gamo and Gofa zone, southern Ethiopia, 2023 (n = 231).

Variable		Frequency	Percent
Position	Head department	15	6.5
	Staff professional	212	91.8
	Management areas	4	1.7
Year of experience (years)	Less than 5	125	54.1
	5 & More than 5	106	45.9
Working time	Part time	21	9.1
	Full time	210	90.9
Computer access at work place	Yes	27	11.7
	No	204	88.3
Internet access at work place	Yes	104	45.0
	No	127	55.0
Training on EBP	Yes	94	40.7
	No	137	59.3
No. of delivery attended per month	< 10	12	5.2
	10–20	102	44.2
	>20	117	50.6
Standard guideline available at work place	Yes	154	66.7
	No	77	33.3

### Knowledge and attitude of recommended intrapartum care practices

The study participants had a mean score of 27.36 (SD ±4.35) and a minimum and maximum score of 13 and 33, respectively, indicating 114 (49.4%) had good knowledge on recommended WHO intrapartum practices. Study participants who knew evidence-based intrapartum care for a positive childbirth experience were 173 (74.9%), and of these, 60.8% (139) participants understood that evidence-based practice means giving care based on WHO guidelines.128 (55.4%) of the obstetric care providers in the survey had a positive attitude. 32.4 (SD ±5.35) was the mean attitude score regarding evidence-based intrapartum treatment. All obstetricians, IESOs (80%), and general practitioners (62%), in order of preference, have strong expertise, according to study participants. As with knowledge, all obstetricians, followed by IESOs (55%), midwives (54.8%), and general practitioners (48.2%), had a positive attitude.

### Magnitude of intrapartum harmful practices

**Fig 2:**
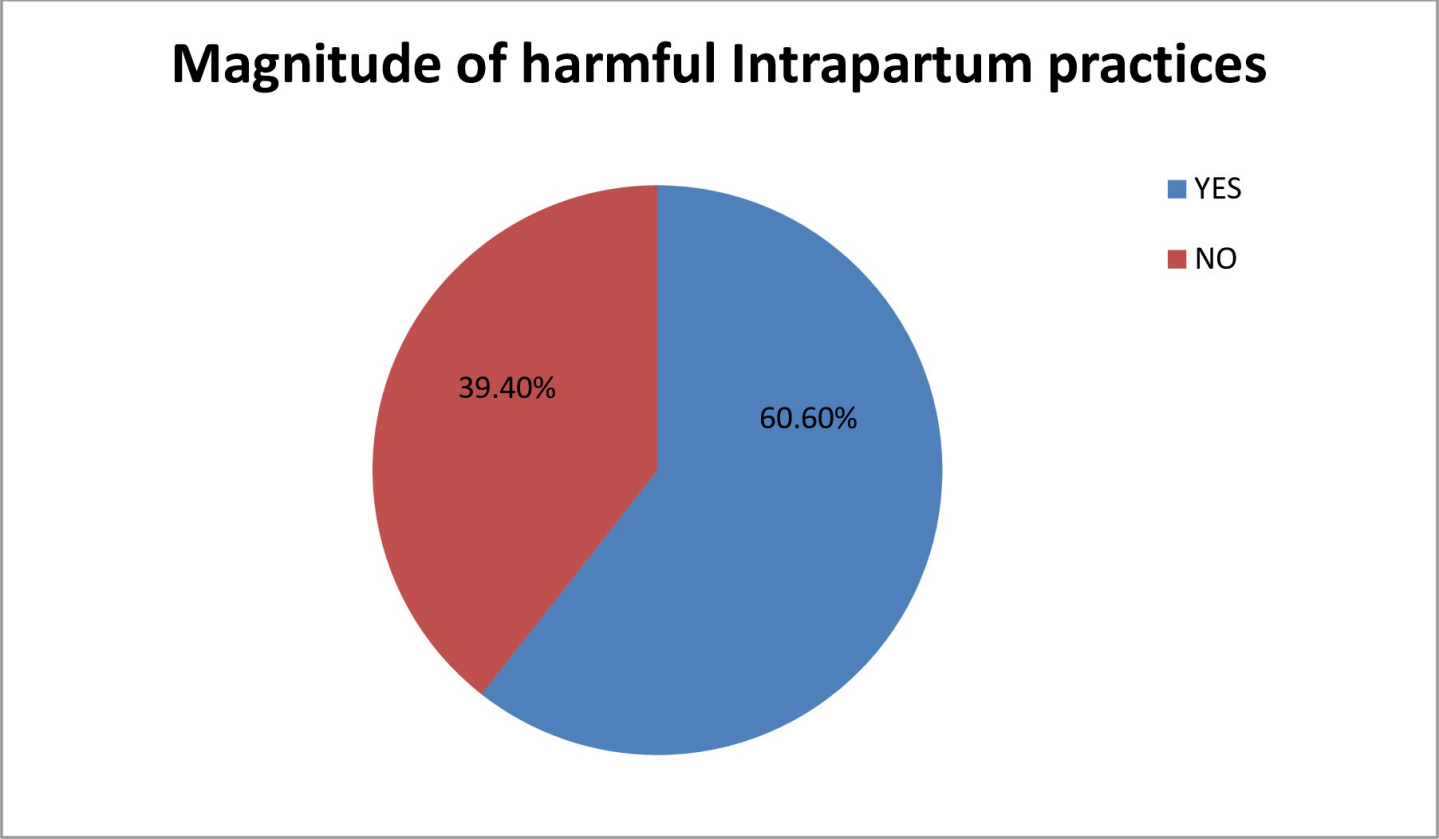
Magnitude of intrapartum harmful practices among obstetric care providers in government hospitals of Gamo and Gofa zone, southern Ethiopia, 2023 (n = 231).

The study assessed the extent of non-recommended intrapartum practices by examining six items related to intra-partum practices not recommended by the WHO. The overall prevalence of harmful intrapartum practices was found to be 60.6% (95% CI = 53.25–68.5) (**Fig 2**).

### Distribution of non-recommended (harmful) intrapartum practices

**Fig 3:**
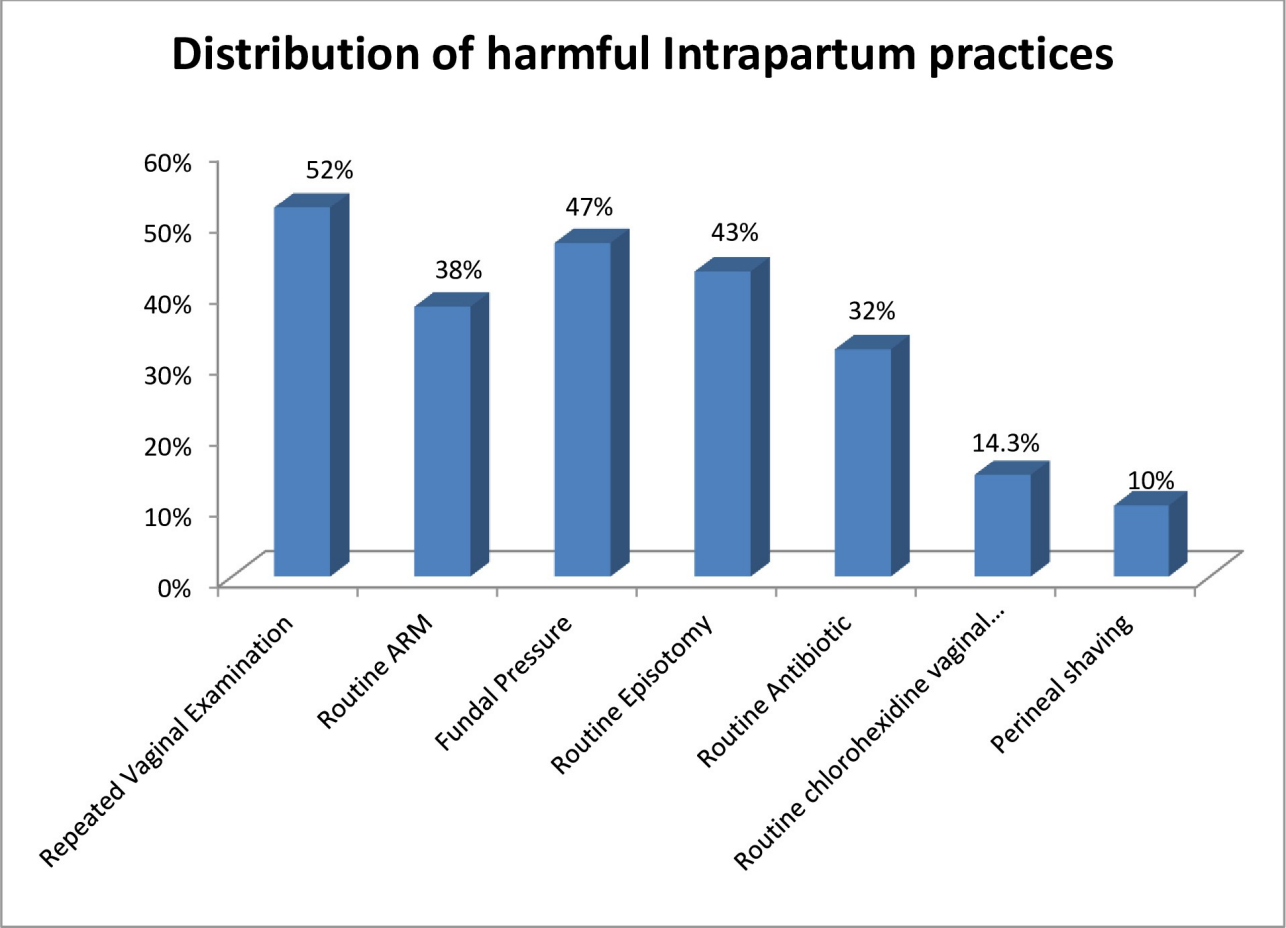
Distribution of non-recommended (harmful) intrapartum practices among obstetric care providers in government hospitals of Gamo and Gofa zone, southern Ethiopia, 2023 (n = 231).

The majority of the respondents practiced harmful intrapartum cares such as fundal pressure during second stage of labour (47%) and repeated per-vaginal examination without indication (52%). The most common practiced non-adherent WHO recommendation was repeated manual vaginal examination (**Fig 3**).

### Factors associated with harmful intrapartum practices

A bivariate analysis was done to identify factors associated with harmful intrapartum care. All variables that have an association with the outcome variables in bivariate analysis (p value <0.25) were included in the multivariate analysis model. Variables that had an association with the outcome variable on bivariate analysis were sex of the professional, educational level, working time, knowledge, attitude of the professional, training on evidence based intrapartum care within 12 months, year of experience, internet access, information on health related issue, knowledge towards harmful practices and having in-service training on intrapartum care.

The multivariable logistic regression analysis revealed that lack of internet access, not being trained on evidence based intrapartum care and less year of experience all had a statistically significant association with the outcome variable, harmful intrapartum practices (Table 3).

**Table 3:** Multivariate logistic regression analysis result for variables associated with harmful intrapartum practices among obstetric care providers in government hospitals of Gamo and Gofa zone, southern Ethiopia, 2023 (n = 231).

Variables		Harmful practice		COR(95%CI)	AOR(95%CI)	P-value
		Yes (%)	No (%)			
Year of experience	< 5 years	98(78.4)	27 (21.6)	5.53 (3.10,9.84)	6.21 (3.1,12.5)	.002*
	≥ 5 years	42 (39.6)	64 (60.4)	1	1	-
Training on EBP	Yes	37(39.4)	57 (61.6)	1	1	
	No	103(75.2)	34 (24.8)	4.6 (2.64,8.23)	4.02 (1.94,7.95)	.000*
Internet accessibility	Yes	35(33.7)	69 (66.3)	1	1	-
	No	105(82.7)	22 (17.3)	9.4 (5.09,17.38)	10.1 (4.9,21.1)	.003*

The odds of practicing harmful intrapartum care among respondents who had been working for less than five years were found to be 6 times higher compared to those who had been working for more than five years (AOR = 6.21, 95% CI (3.1–12.5). Similarly, those obstetric care providers who had no received training on evidence based intrapartum cares have 4 times higher likelihood of practicing non-recommended intrapartum cares than its counterparts (AOR = 4.01, 95% CI: 1.94–7.95). Additionally, the odds of practicing harmful intrapartum care among respondents who had no sufficient internet access were found to be 10 times higher (AOR = 10.1, 95% CI: 4.93–21.1).

## Discussion

The overall magnitude of harmful intrapartum care in southern Ethiopia was found to be 60.6%. The majority of the respondents practiced harmful intrapartum cares such as fundal pressure during second stage of labour (47%). The proportion of harmful intrapartum practice in this study is relatively consistent with studies conducted in Arba Minch district, Gamo Gofa zone, Southern Ethiopia This However this finding is higher than studies conducted in Philippe [30], south Wollo zone [27], which, was 39.4%, northwest Ethiopia, 27.5% [10] and Addis Ababa, Ethiopia [15] the reasons for performing fundal pressure might be to improve fetal head descending is during long or prolonged 2nd stage of labour and weak maternal pushing effort. Because of trust in its effectiveness, fundal pressure was often selected as the first option to hasten the second stage of labour to avoid vacuum extraction or caesarean section.

Another most common distribution of non-recommended (harmful) intrapartum care was repeated per-vaginal examination without indication and less than four hours was 52%. This finding was consistence with study conducted in northwest Ethiopia [10]. This might be due to similarity in the data collection tool, study design, study participants, and the closeness of the study period. Furthermore, the third common practiced harmful, non-recommended intrapartum care was routine episiotomy. Short-term problems from episiotomy can be excruciating at times, but there are also long-term effects that can seriously interfere with day-to-day activities. Short-term issues may consist of: Perineal laceration: the actual incision Dehiscence of episiotomy (separation of the wound). This magnitude is lower than study conducted in northwest, Amhara region, Ethiopia [10].

In this study, there was a strong correlation between harmful intrapartum care and the obstetric care providers’ personal experiences (AOR = 6.21, 95% CI (3.1–12.5). The odds of practicing harmful intrapartum care among respondents who had been working for less than five years were found to be 6 times higher compared to those who had been working for more than five years. This could be because medical professionals who have worked for a number of years are better at handling patients because they have seen similar instances frequently and have learned from mistakes made [31, 32]. Additionally, because they had training that gave them access to up-to-date intrapartum health knowledge, obstetric care professionals with multiple years of experience may be more motivated to practice. This finding was supported by studies conducted in Punjab, Pakistan [33] and Addis Ababa, Ethiopia [15].

Similarly, those obstetric care providers who had no received training on evidence based intrapartum cares have 4 times higher likelihood of practicing non-recommended intrapartum cares than its counterparts (AOR = 4.01, 95% CI: 1.94–7.95). This finding was inconsistent with study from Africa setting [34]. However, this figure is in line with studies from Kenya [35] and south Wollo, Ethiopia [27]. The reason for this could be that obstetric care providers who have received training in recommended and recently discovered scientifically based intrapartum care may have begun implementing new evidence.

Additionally, the odds of practicing harmful intrapartum care among respondents who had no sufficient internet access were found to be 10 times higher (AOR = 10.1, 95% CI: 4.93–21.1). This suggests that obstetric care professionals who had access to internet may be aware of how to apply research findings to clinical practice if they had used intrapartum-related health information to update their knowledge. Additionally, their regular reading habits may serve to drive them to apply new data. This find was supported be different studies from Cameron, Iceland and Ethiopia [27, 34, 36].

In general, the study’s implications are highly pertinent because Ethiopian intrapartum practices are unknown to be detrimental. Moreover, policymakers may utilize this study as a pillar to reform healthcare providers.

### Limitation of study

The discussion in the study lacks comparability due to inadequate literature reviews on similar study topics. Moreover, the observed effects might be influenced by the Hawthorne effect. Additionally, the study did not provide information on the degree or severity of occurrences of harmful intrapartum care practices. Instead, instances of harmful practices were consolidated into a binary variable, categorized as either "Yes" or "No." On the other hand, in this study, both obstetric providers who engaged in multiple non-recommended care practices and those who committed a single harmful practice were treated equally and counted in the same manner (assigned as “Yes”).

## Conclusion

The findings of this study implicated that more than half of obstetric care providers practiced non-recommended (harmful) intrapartum cares. Lack of training on intrapartum practice, lack of sufficient internet access, and work experience were associated with the harmful intrapartum practice.

## Supporting information

S1 File Tool file. (DOCX)

S2 File SPSS file. (SAV)
