# Supplementary material for: Observation of non-recommended (harmful) intrapartum practices among obstetric care providers in public hospitals in southern Ethiopia, 2023
Source: PLOS Glob Public Health. 2024 Jul 11;4(7):e0003375. doi: 10.1371/journal.pgph.0003375 (PMC11239106; doi:10.1371/journal.pgph.0003375)
Supplement: S1 File — (DOCX) [file pgph.0003375.s001.docx]

# QUESTIONNAIRES

**Part I: Socio-Demographic Information**

| S.no | Question | Answers | Remark |
| --- | --- | --- | --- |
| 101 | Age | _____________in years |  |
| 102. | Sex: | 1. Female  2. Male |  |
| 103. | Profession: | 1. Medical doctor  2. Midwife  3 Nurse  4. IESO  5.Gynecologist |  |
| 104 | Educational level: | 1. GP 2. MSc 3. Bachelor Degree 4. Diploma nurse 5. Diploma midwife |  |
| 105 | Marital status | 1. Single 2. Married 3. Divorced 4. Widowed |  |
| 106 | Monthly salary (ETB) | __________________ |  |

**Part II: Organization and individual related information**

| S.no | Question | Answer | Remark |
| --- | --- | --- | --- |
| 201 | Year of experience: | _____________in years |  |
| 202 | Qualification | 1. Head department 2. Staff midwifes 3. Management area 4. Other, specify__ |  |
| 203 | In which working time do you work here | 1. Part time 2. Full time |  |
| 204 | Do you have access to a computer at your workplace? | 1. Yes  2. No |  |
| 205 | If you say yes to question number 204, for what purpose do you use the computer? | 1. for scientific reading  2. For digital communication  3. For patient data documentation |  |
| 206 | Do you have access to internet at your work place? | 1. Yes  2. No |  |
| 207 | If you say yes to question number 206, what do you search on the internet commonly? | 1. Online journals  2. Social networks  3. Cochrane Database  4.WHO RHL |  |
| 208 | Do you have access to updated/standard intrapartum care guideline at your work place? | 1. Yes 2. No |  |
| 209 | If yes for question no- 208, which patient care guideline is available at your work facility | 1. WHO safe child birth checklist 2. BEmONC patient care manual 3. FMOH intrapartum care protocol |  |
| 210 | What resources do you use to access health information? | 1. Scientific journals  2. Text books  3. Colleagues  4.Conferences |  |
| 211 | What motivates you to seek health information at your work place? (You can choose more than one) | 1. Managerial support  2. Role clarity  3. Scientific meetings  4. Patient’s condition  5. Skill building workshops  6. Regular mentorship  7. Cooperation among staff members |  |
| 212 | In the past six month, have you participated in any of the following professional activities related to your area of work? | 1. conference  2. Training on evidence based practice  3. Seminar  4. Case presentation  5. Never at all |  |
| 213 | Have you taken in-service training in intra-partum care? | 1. Yes  2. No |  |
| 214 | How many delivery do you attend in a month | ______________ |  |

**Part III: Knowledge questions**

| 301 | Do you know evidence based intra-partum care for a positive child birth experience? | 1. Yes 2. No | | | If “No,, go to 303 |
| --- | --- | --- | --- | --- | --- |
| 302 | If you answered yes, what do you understand by evidence- based care? (you can select more than one) | 1. Patient preference and value based care 2. Expert’s opinion and experience based 3. Using WHO updated guideline 4. Research findings based | | |  |
| 303 | Does giving care in accordance with the WHO guideline improve quality of intrapartum care? | 1. Yes 2. No | | |  |
| 304 | Do you know obstetrical cares that are recommended and not recommended / potentially harmful during labour and delivery? | 1. Yes 2. No | | | If “No,, go to 307 |
| 305 | If yes for question number 304, which practices are recommended to during labour and delivery? (Please encircle 1 as many as you think, and 2 if you think no effect) |  | Yes | No |  |
|  |  | Respectful maternal care | 1 | 2 |  |
|  |  | Effective communication | 1 | 2 |  |
|  |  | Companionship during labor | 1 | 2 |  |
|  |  | Continuity of care | 1 | 2 |  |
|  |  | Oral fluid and food | 1 | 2 |  |
|  |  | Encouraging the adoption of mobility and an upright position during labour | 1 | 2 |  |
|  |  | encouraging the adoption of a birth position of the individual woman’s choice, including upright positions | 1 | 2 |  |
|  |  | perineal massage, warm compresses and a “hands on” guarding of the perineum for prevention of perineal tear | 1 | 2 |  |
|  |  | Use of Prophylactic uterotonic for prevention of PPH | 1 | 2 |  |
|  |  | Delayed umbilical cord clamping | 1 | 2 |  |
|  |  | Controlled cord traction |  |  |  |
|  |  | Auscultation using a Doppler ultrasound device or Pinard fetal stethoscope during admission | 1 | 2 |  |
|  |  | Digital vaginal examination at intervals of four hours | 1 | 2 |  |
|  |  | Intermittent auscultation of the fetal heart rate with either a Doppler ultrasound device or Pinard fetal stethoscope | 1 | 2 |  |
|  |  | Postpartum abdominal uterine tonus assessment | 1 | 2 |  |
|  |  | All newborns should be given 1 mg of vitamin K intramuscularly | 1 | 2 |  |
|  |  | Newborns without complications should be kept in skin-to-skin contact | 1 | 2 |  |
|  |  | All newborns, who are able to breastfeed, should be put to the breast as soon as possible after birth | 1 | 2 |  |
| 306 | If you say yes for question number 304, which interventions are not recommended or potentially harmful in a laboring mother? (Please circle ‘1’ if practice is non- recommended and ‘2’ for Not) |  | Yes | No |  |
|  |  | Routine clinical pelvimetry on admission in labour | 1 | 2 |  |
|  |  | Routine cardiotocography for the assessment of fetal well-being on labour admission | 1 | 2 |  |
|  |  | Routine perineal/pubic shaving prior to giving vaginal birth | 1 | 2 |  |
|  |  | Administration of enema for reducing the use of labour augmentation | 1 | 2 |  |
|  |  | Continuous cardiotocography for assessment of fetal well-being | 1 | 2 |  |
|  |  | Routine vaginal cleansing with chlorhexidine during labour for the purpose of preventing infectious morbidities | 1 | 2 |  |
|  |  | Routine or liberal use of episiotomy | 1 | 2 |  |
|  |  | Application of manual fundal pressure to facilitate childbirth during the second stage of labour | 1 | 2 |  |
|  |  | Routine antibiotic prophylaxis for women with episiotomy |  |  |  |
|  |  | The use of routine amniotomy alone for prevention of delay in labour |  |  |  |
|  |  | Routine nasal or oral suction of newborn | 1 | 2 |  |

**Part IV: Attitude questions**

| 401 | Good communication and support to laboring mother facilitates progress of labor and improves birth outcome. | Strongly agree | 5 |  |
| --- | --- | --- | --- | --- |
|  |  | Agree | 4 |  |
|  |  | Neutral | 3 |  |
|  |  | Disagree | 2 |  |
|  |  | Strongly disagree | 1 |  |
| 402 | Ambulation and freedom of movement in labour are safe, more satisfying for women, and facilitate the progress of labour. | Strongly agree | 5 |  |
|  |  | Agree | 4 |  |
|  |  | Neutral | 3 |  |
|  |  | Disagree | 2 |  |
|  |  | Strongly disagree | 1 |  |
| 403 | Fundal pressure used during the second stage of labour predispose for uterine rupture. | Strongly agree | 5 |  |
|  |  | Agree | 4 |  |
|  |  | Neutral | 3 |  |
|  |  | Disagree | 2 |  |
|  |  | Strongly disagree | 1 |  |
| 404 | Intravenous infusions should not be used for every laboring mother as a way of rehydration. | Strongly agree | 5 |  |
|  |  | Agree | 4 |  |
|  |  | Neutral | 3 |  |
|  |  | Disagree | 2 |  |
|  |  | Strongly disagree | 1 |  |
| 405 | Continuous labor support should be the standard of care for all laboring women. | Strongly agree | 5 |  |
|  |  | Agree | 4 |  |
|  |  | Neutral | 3 |  |
|  |  | Disagree | 2 |  |
|  |  | Strongly disagree | 1 |  |
| 406 | Frequent vaginal examination predispose to maternal and newborn infection. | Strongly agree | 5 |  |
|  |  | Agree | 4 |  |
|  |  | Neutral | 3 |  |
|  |  | Disagree | 2 |  |
|  |  | Strongly disagree | 1 |  |
| 407 | Labor and delivery care given based on client preference improves clients service seeking. | Strongly agree | 5 |  |
|  |  | Agree | 4 |  |
|  |  | Neutral | 3 |  |
|  |  | Disagree | 2 |  |
|  |  | Strongly disagree | 1 |  |
| 408 | There is no evidence to support routine episiotomy and aggressive perineal massage or retracting at birth. | Strongly agree | 5 |  |
|  |  | Agree | 4 |  |
|  |  | Neutral | 3 |  |
|  |  | Disagree | 2 |  |
|  |  | Strongly disagree | 1 |  |

**OBSERVATION CHECKLIST**

Part V: observation checklist of practice of evidence based intrapartum care (circle 1 if OCP practiced the listed activity and 2 if not); to be observed three times with copied checklist.

| **Non-recommended intrapartum practices** | | |
| --- | --- | --- |
| 517 | Fundal pressure in second stage of labor | 1. Yes 2. No |
| 518 | Routine episiotomy | 1. Yes 2. No |
| 519 | Routine IV fluids for prevention of labor delay | 1. Yes 2. No |
| 520 | Routine amniotomy to prevent labor delay | 1. Yes 2. No |
| 521 | Routine vaginal cleansing with chlorohexidine during labour | 1. Yes 2. No |
| 522 | perineal/pubic shaving prior to giving vaginal birth | 1. Yes 2. No |
| 523 | Routine antibiotic prophylaxis | 1. Yes 2. No |
| 524 | Continuous CTG without indication | 1. Yes 2. No |

Thank you very much for taking time to answer these questions. I appreciate your help.
